# Ultrasound cyclo-plasty for moderate glaucoma: Eighteen-month results from a prospective study

**DOI:** 10.3389/fmed.2022.1009273

**Published:** 2022-12-16

**Authors:** Rui-Xue Wang, Ning Li, Xiao-Ya Chen

**Affiliations:** ^1^The Affiliated Xuzhou Municipal Hospital of Xuzhou Medical University, Xuzhou, Jiangsu, China; ^2^Department of Ophthalmology, Xuzhou First People’s Hospital, Xuzhou, Jiangsu, China; ^3^Department of Ophthalmology, The First Affiliated Hospital of Anhui Medical University, Hefei, Anhui, China

**Keywords:** glaucoma, ultrasound cyclo-plasty, intraocular pressure, ciliary body, treatment

## Abstract

**Purpose:**

To evaluate the long-term clinical efficacy of ultrasound cyclo-plasty (UCP) in the treatment of moderate glaucoma and molecular effects in animal experiments.

**Methods:**

An 18-month clinical study was conducted among 32 patients with moderate glaucoma. The primary outcome was surgical success, defined as a reduction in intraocular pressure (IOP) of greater than or equal to 20% from the baseline and an IOP value of greater than 5 mmHg at the last follow-up. The secondary outcomes were related to the quality of life, complications, and mean IOP value at each follow-up. In the animal experiment, 20 New Zealand rabbits were used to establish a high-IOP model and implement UCP. The distribution of aquaporin 4 (AQP4) in the ciliary body and the tissue changes under electron microscopy were observed after surgery.

**Results:**

The mean patient IOP decreased from 34.9 ± 4.9 mmHg before surgery to 23.5 ± 5.2 mmHg at 18 months after UCP. No vision loss occurred in any patient. Some patients had postoperative complications, but the symptoms were mild and disappeared within 3 months after the surgery. Most patients had good postoperative quality of life. Histology showed that AQP4 remained in the ciliary muscle after UCP, and only the bilayered epithelial cells showed coagulative necrosis. Furthermore, electron microscopic observation revealed the destruction of ciliary process cells covered by ultrasound after UCP.

**Conclusion:**

UCP is associated with mild postoperative reactions and the mild treatment of ciliary tissue and is a safe and effective method for reducing IOP in moderate glaucoma.

## Introduction

Glaucoma is a progressive optical neuropathy caused by the accelerated degeneration of retinal ganglion cells. This degeneration severely threatens and impairs the visual pathway, eventually leading to blindness (1, 2). Therefore, it is essential to control the progression of early and moderate glaucoma and effectively protect the remaining vision. Currently, lowering the intraocular pressure (IOP) is the most important therapeutic measure to prevent and delay diminution of vision. This can be achieved clinically by reducing the inflow or increasing the outflow of the aqueous humor (1, 3, 4). Surgical destruction of the ciliary body can reduce the secretion of aqueous humor. The traditional surgical methods include cyclocryotherapy, cyclodiathermy, and diode laser ring photocoagulation (5, 6). However, due to the difficulties in precisely selecting the target ciliary body and determining the appropriate dosage, surgery often leads to damaged adjacent tissue and ocular inflammation. This increases the incidence of postoperative complications and hinders the accurate prediction of curative effects.

In recent years, the development of high-intensity focused ultrasound (HIFU) technology has advanced significantly. As a new type of non-invasive annular destruction surgery, ultrasound cyclo-plasty (UCP) can effectively reduce IOP (7). In comparison with traditional surgery, the ultrasound energy is focused using a non-optical transparent medium. The energy deposition and tissue heating at the focus are independent of cytochrome deposition and can be arbitrarily located in the intraocular tissue, thus selectively destroying the ciliary body (8, 9). The advantage of focused ultrasound is that it can focus the energy within a suitable range of tissue, and the volume of tissue destroyed is preset by the machine to avoid excessive damage (10). Thus far, many studies have shown that UCP is an effective and well-tolerated method for reducing IOP, but its therapeutic effects on moderate glaucoma are still being explored. The purpose of the present study was to examine the molecular effects and clinical efficacy of UCP for the treatment of moderate glaucoma (11).

## Materials and methods

### High-intensity focused ultrasound equipment

The study used an EyeOP1 device imported from France, which has been described in detail previously (12). In short, the EyeOP1 consists of a command module and a therapy device; it generates voltage through a signal generator and then raises the voltage to a certain level via an amplifier to ensure that the ultrasonic beam is emitted. The instrument’s ring-shaped treatment probe contains 6 miniature piezoelectric transducers, and the contact surface is arc-shaped and rectangular. During the treatment, it acts on each sector of the ciliary body while avoiding the nasal-temporal meridian (8, 13). To stably center the probe, the suction on the bottom of the positioning ring applies a vacuum at a low level, bringing it into closer contact with the eye. The design of the probe is unique and innovative. The probe is available in 3 diameters (11 mm, 12 mm, and 13 mm), and the appropriate diameter can be selected according to the eye conditions of each individual patient.

### Patients

This study was conducted in accordance with the principles of the Declaration of Helsinki and ISO 14155 standard and was approved by the local institutional review board. Written content was obtained from all enrolled patients.

This study included 32 eyes of 32 patients with moderate glaucoma who underwent UCP. The glaucoma staging was performed by glaucoma specialist based on the Hoddap -Parrish-Anderson criteria (14).

The inclusion criteria were as follows: (1) Patients diagnosed with moderate primary open angle glaucoma, (2) IOP not controlled by hypotensive medication, (3) IOP greater than or equal to 20 mmHg, (4) age more than 18 years old and less than 90 years old, (5) patients who signed the informed consent and who were able to complete all postoperative follow-up visits, (6) the type and amount of ocular hypotensive medication remained the same before and after treatment.

The exclusion criteria were as follows: (1) Eye infection in any eye in the 2 weeks before treatment, (2) any medical or treatment history or systemic disease that may affect the evaluation of the treatment efficacy, (3) pregnant or lactating women, (4) patients who underwent other procedures at the same time, and (5) patients who underwent other eye surgeries for reducing IOP within 18 months after the surgery.

**FIGURE 1:**
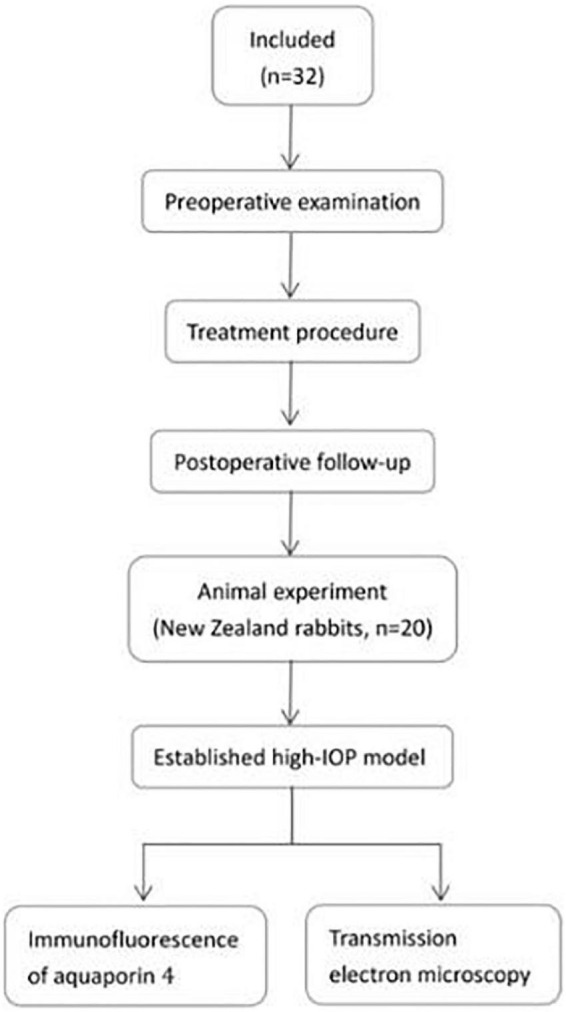
Schematic diagram of the research flowchart.

A schematic diagram of the research flowchart is shown in Figure 1.

### Preoperative examination

Each patient underwent a routine eye examination, which included uncorrected visual acuity, photography of the anterior segment, gonioscopy, perimetry, fundus photography, IOP (Goldman tonometer), ultrasound biomicroscopy (UBM), biological parameters of the eyeball (axial length, white-to-white distance), and analysis of the quality of life.

### Treatment procedure

Both anesthesia and treatment were performed by the same experienced ophthalmologist. Retrobulbar anesthesia was administered in all patients. The surgeon selected the appropriate treatment procedure according to each patient’s conditions to accurately determine the ultrasound dosage. Since the subjects of this study were patients with moderate glaucoma and no patient had an IOP of more than 36 mmHg, all patients were treated with 6 sectors and the exposure time was 8 s.

The specific steps were as follows. First, the patient lay supine, with the eye axis perpendicular to the horizontal line. After the instrument was started, the positioning cone was adsorbed on the ocular surface and centered. Second, the negative pressure suction was activated and filled with saline solution, stepped on the pedal to start the treatment. Finally, after the treatment, the probe and positioning cone were removed. The patients stayed in the hospital for observation for 2 h.

Tobramycin dexamethasone eye drops were added within 4 weeks after surgery, 4 times a day.

### Postoperative follow-up

Follow-up visits occurred 1 day, 1 week, 1, 3, 6, 12, and 18 months after treatment. Eye examinations such as uncorrected visual acuity, photography of the anterior segment, IOP (Goldman tonometer), complication assessment, and quality of life analysis were performed at each visit.

### Outcome measures

The surgical success criteria were an IOP reduction of more than or equal to 20% compared with the baseline value and an IOP of more than 5 mmHg at the last follow-up visit, without adding new glaucoma medication compared to baseline.

The Glau-QoL 36-item questionnaire was used to assess the patients’ quality of life, with 7 domains: psychological wellbeing, self-image, daily life, burden of treatment, driving, anxiety, and confidence in health care. Each item had 3–9 questions, with each question’s response collected on a 5-point scale. Each item was scored separately; a patient’s score was the sum of the total scores of all questions, transformed into a scale from 0 to 100.

### Animal experiment

All animal experiments have been approved by the ethics Committee of Xuzhou First People’s Hospital. All animal testing methods were carried out in compliance with the ARRIVE guidelines.

A high-IOP model was established for 20 New Zealand rabbits. Puncture at the corneal margin, extract 0.1–0.2 ml of aqueous humor, and then inject an equal amount of compound carbomer. At 2 weeks, rabbit high IOP is basically stable. Each New Zealand rabbit was injected into the ear vein with 10% chloralhydrate (3.5 mg/kg) intravenously for general anesthesia, and one eye was treated with UCP and the other eye was used as a negative control. The rabbits were executed immediately after the treatment. Both eyeballs were removed, and the ciliary bodies were separated.

#### Immunofluorescence of aquaporin 4

The ciliary body tissue was fixed in 4% paraformaldehyde solution and embedded in paraffin to make 5-μm-thick sections. The tissue sections were rinsed with phosphate-buffered saline (PBS) for 30 min, treated with formaldehyde-H_2_O_2_ for 10 to 15 min, treated with Triton X-100 for 10 min, and blocked with normal goat serum for 4 h. After drying, the primary antibody (AQP4, 1:400) was added, and the samples were incubated overnight at 37°C. Next, the samples were rinsed with PBS, and secondary antibody (Cy3-labeled goat anti-rabbit IgG) was added. After 90 min, the samples were rinsed with PBS. Then, slides were mounted and observed under a laser confocal microscope.

#### Transmission electron microscopy

The ciliary body tissue was fixed in 2.5% glutaraldehyde solution, rinsed with 0.1 M phosphate buffer, fixed with 1% osmic acid solution, and rinsed again. This was followed by gradient dehydration and epoxy resin immersion embedding. Ultra-thin sections (70 nm) were obtained using an ultra-thin cutting machine (UC7, Leica, Solms, Germany), stained with uranyl acetate and lead citrate solution, and observed under a transmission electron microscope (CM120, Philips Electronics, Mahwah, NJ, USA).

### Statistical analysis

Data were analyzed using SPSS 23.0 statistical software (IBM, USA). Enumeration data, such as gender and lens status, were expressed as cases. Measurement data were expressed as means ± standard deviations (x ± s). The Wilcoxon rank-sum test was used to compare the differences between the IOP and quality of life scores during follow-up and the baseline values. The level of statistical significance was set at *p*<0.05.

## Results

### Patient characteristics

All 32 patients completed the surgery successfully. The patient details are described in Table 1.

**TABLE 1:** Patients characteristics.

Patients	32
Age, mean ± SD, year	56.8 ± 10
**Sex**	
Male	17
Female	15
BCVA, logMAR	0.76 ± 0.31
IOP baseline, mean ± SD	34.9 ± 4.9
Axial length	23.77 ± 0.80
White to white	11.74 ± 0.17
**Lens status**	
Phakic	29
Pseudophakic	3
Aphakic	0
Preoperative hypotensive medications, mean ± SD	1.7 ± 0.7

BCVA, best-corrected visual acuity; IOP, Intraocular pressure; SD, Standard deviation.

### Intraocular pressure

**FIGURE 2:**
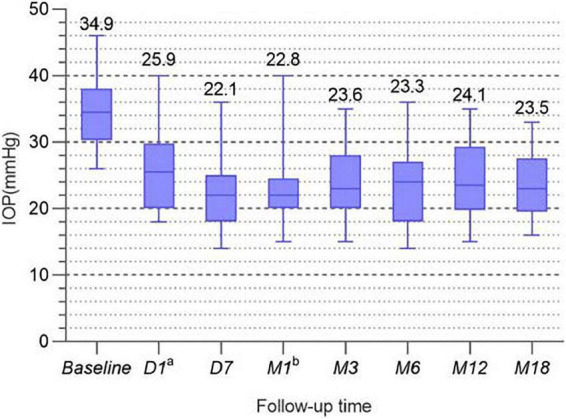
The mean IOP of all patients.

At all follow-up visits, the mean IOP value for each measurement decreased significantly from the baseline value. The mean preoperative IOP was 34.9 ± 4.9 mmHg; 18 months after treatment, the IOP had decreased by 32.6%, and the success rate was as high as 81%. The specific IOPs for the patients and the success rate of the surgery are shown in Table 2. In order to more clearly reflect the IOP trend, Figure 2 shows the line chart of IOP in this study.

**TABLE 2:** Intraocular pressure at baseline and during follow-up in the patients.

	Mean ± SD IOP (no patients)	Relative IOP reduction (%)	Success rate (%)	P*-value compared with the baseline
Baseline	34.9 ± 4.9 (32)	NA	NA	NA
Day 1	25.9 ± 5.6 (32)	25.9	68.8	0.000
Day 7	22.1 ± 5.1 (31)	36.7	87.1	0.000
Month 1	22.8 ± 5.4 (29)	34.7	86.2	0.000
Month 3	23.6 ± 5.4 (28)	32.3	78.6	0.000
Month 6	23.3 ± 6.1 (29)	33.3	82.8	0.000
Month 12	24.1 ± 6.0 (26)	31.0	76.9	0.000
Month 18	23.5 ± 5.2 (21)	32.6	81.0	0.000

*Wilcoxon test. NA, not applicable; IOP, Intraocular pressure; SD, Standard deviation.

### Complications

The complications of UCP were categorized as intraoperative and postoperative. Four patients had mild pain during the operation. The postoperative complications were also mild, and most of them resolved spontaneously within 1 month. The types and numbers of complications are shown in Table 3.

**TABLE 3:** Intra-operative and post-operative complications.

Description	Number of eyes (%)
**Intraoperative**	
Pain	4 (12.5%)
**Post-operative**	
Loss of visual acuity (>2 lines)	1 (3.1%)
Induced astigmatism (>1 diopter)	1 (3.1%)
Conjunctival hyperemia	3 (9.4%)
Subconjunctival hyperemia	1 (3.1%)
Corneal edema	2 (6.3%)
Superficial punctate keratitis	1 (3.1%)
Hyphema	1 (3.1%)
Aqueous flare (<7 days)	5 (15.6%)
Retinal detachment	0 (0%)
Induced cataract	0 (0%)
Phthisis	0 (0%)

### Quality-of-life analysis

**FIGURE 3:**
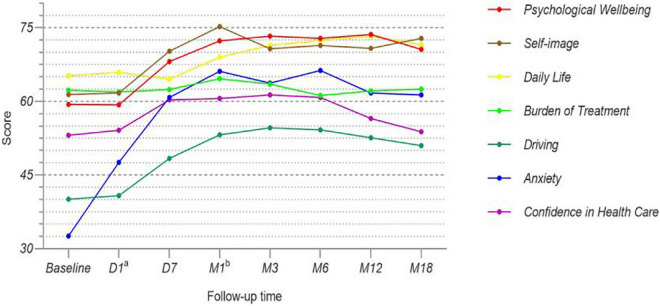
Each survival index in all patients.

All patients completed the questionnaire once preoperatively and again at each postoperative follow-up visit. We analyzed the trends in each survival index, in detail, through calculations and scoring. All of the scores are shown in Table 4. In order to get a better overview of the patients’ quality of life, we made a line chart, with each indicator marked in a different color (Figure 3).

**TABLE 4:** Score distribution of the health-related quality of life domains for patients.

	Score							
	
		Mean ± SD						
		
	Number of patients (MD)	Psychological wellbeing	Self-image	Daily life	Burden of treatment	Driving	Anxiety	Confidence in health care
Baseline	32 (0)	59.4 ± 17.6	61.4 ± 20.8	65.2 ± 21.1	62.3 ± 17.1	40.1 ± 25.6	32.6 ± 21.1	53.1 ± 16.7
Day 1	29 (3)	59.3 ± 18.2	61.7 ± 19.8	65.9 ± 20.8	61.9 ± 16.3	40.8 ± 25.8	47.6 ± 20.1*	54.1 ± 17.9
Day 7	29 (3)	68.1 ± 16.5*	70.2 ± 19.1*	64.5 ± 20.3	62.4 ± 18.7	48.4 ± 23.4*	60.8 ± 21.9*	60.3 ± 16.1*
Month 1	26 (6)	72.3 ± 13.1*	75.2 ± 16.8*	69.0 ± 18.2*	64.6 ± 16.7	53.2 ± 26.8*	66.1 ± 24.3*	60.6 ± 16.6*
Month 3	27 (5)	73.3 ± 14.1*	70.7 ± 16.5*	71.4 ± 18.2*	63.5 ± 17.4	54.6 ± 26.1*	63.7 ± 20.9*	61.3 ± 14.8*
Month 6	25 (7)	72.8 ± 14.2*	71.4 ± 16.6*	72.4 ± 14.8*	61.2 ± 16.9	54.2 ± 20.9*	66.3 ± 19.0*	60.8 ± 14.6*
Month 12	24 (8)	73.6 ± 12.1*	70.8 ± 18.9*	73.2 ± 13.9*	62.1 ± 17.6	52.6 ± 20.6*	61.7 ± 20.6*	56.5 ± 16.2
Month 18	20 (12)	70.6 ± 11.6*	72.8 ± 17.7*	71.5 ± 15.8*	62.5 ± 14.1	51.0 ± 24.7*	61.3 ± 21.7*	53.8 ± 16.5

*P < 0.05. MD, Missing data, SD, Standard deviation.

### Aquaporin 4 expression

**FIGURE 4:**
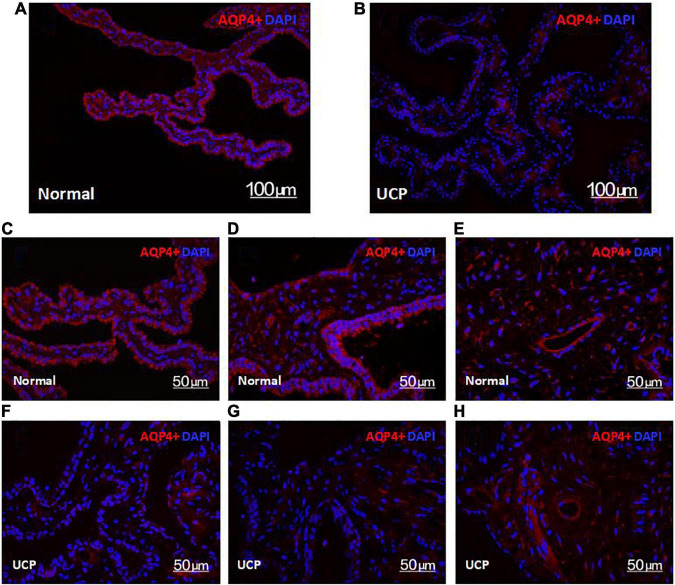
Confocal microscopy images of the expression of AQP4 in the normal ciliary body **(A)** and UCP postoperative **(B)**. Scale bars 100 μm. **(C–G)** are ciliary, processes, **(E,H)** are ciliary, muscles. Scale bars 50 μm.

AQP4 exists in the ciliary body tissue, controls the rate of the formation of aqueous humor by promoting its secretion and absorption, and plays a role in the aqueous balance and pressure regulation of ocular tissues. Therefore, observing the expression of AQP4 in the ciliary body helps to clarify the surgical effect. HIFU mainly targets the epithelial cells in the ciliary body, causing coagulation necrosis due to a thermal effect in the affected area. Compared with the control group, the level of AQP4 in the ciliary process was significantly reduced after UCP treatment (Figures 4A,B). As seen in Figures 4C,D,F,G ca 50-μm local map—the UCP group also had relatively less AQP4 distal to the ciliary body. AQP4 was still present in the ciliary muscle, and only the AQP4 part of the epithelial layer was reduced (Figures 4E,H).

### Transmission electron microscopy

**FIGURE 5:**
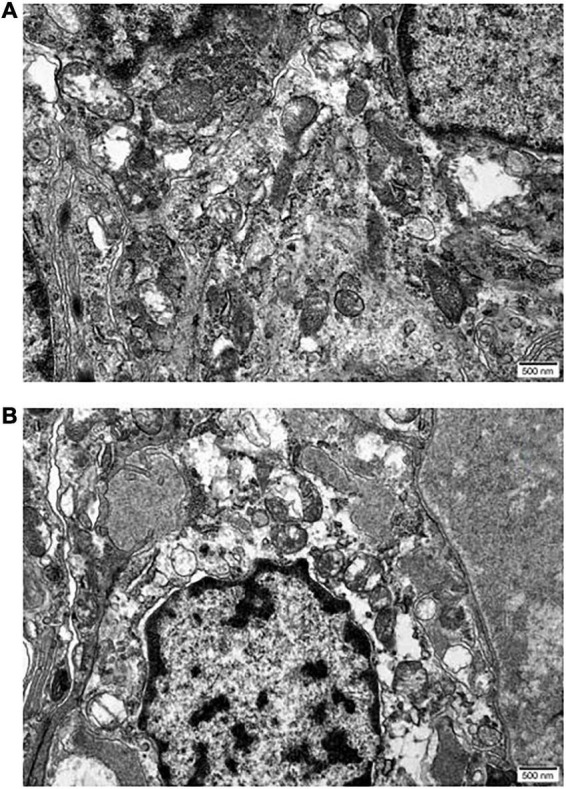
Micrographs reveal the differences between epithelia of normal ciliary body **(A)** and UCP postoperative **(B)**. Scale bars 500 nm.

The internal structure of the ciliary body epithelial cells in the UCP and control groups was observed by transmission electron microscopy. In the untreated epithelial cells, the nuclei were morphologically full with nuclear membrane wrapping, and the mitochondrial structure was basically intact (Figure 5). In contrast, in the UCP group, the nuclei were pyknotic, the nuclear membranes were widened, the chromatin was concentrated and aggregated, and some chromatin was collected under the nuclear membrane and border set occurred. At the same time, the mitochondria were partially destroyed and had become larger and rounder due to swelling, causing cell edema. In addition, the reduction of ribosomes was seen in the epithelial cells after UCP treatment compared with the control group.

## Discussion

Although UCP is generally considered a new technology for the treatment of glaucoma, currently, it is mainly used in patients with advanced to terminal-stage disease, and the effectiveness and safety of the treatment in early and moderate glaucoma are still being explored (15). This study showed that UCP had a remarkable effect on reducing IOP in patients with moderate glaucoma, and the success rate was high. Compared with the traditional annular destruction surgery, UCP may be a step forward in the non-invasive treatment of glaucoma.

During the 18-month follow-up, we recorded the IOP values at each visit, in detail. We observed a 32.6% reduction at the last follow-up—a significant reduction from the preoperative mean (34.9 ± 4.9 mmHg) to 23.5 ± 5.2 mmHg—indicating successful IOP control. The average IOP at each follow-up visit decreased in comparison with the baseline value; these differences were statistically significant. This ideal result was due to the selective necrosis of ciliary body epithelial cells determined by the energy focusing of UCP. In this procedure, sufficient energy can be concentrated within a specific target volume, with sub-millimeter precision. The HIFU device performs annular thermal coagulation of targeted tissue through 6 cylindrical high-frequency sensors (16–18). Six ultrasound beams enter the distal end of the cornea and focus on a larger posterior plane, thereby reducing the secretion of aqueous humor and reducing the pressure of the aqueous humor on the eye wall (18, 19). Additionally, compared to the laser focal effect of diode laser ring photocoagulation, the cylindrical surface can better adapt to the geometry of the target organ while expanding the impact on the ciliary body volume, increasing the treatment area and ensuring the destruction of a sufficient number of ciliary bodies to avoid the rebound elevation of IOP (15, 20). According to our criteria for surgical success, we found that except for the first day after surgery, the average degree of IOP reduction in other follow-up results was more than 30%, and more than 70% of patients achieved success. This may be because in UCP, the destruction of the distal end of the ciliary body is not able to exert its full effect in such a short period of time (19, 21).

A small number of patients felt mild pain during the operation, which may have been related to the patients’ different sensitivities to anesthetic drugs; it may also have been due to the psychological effects of patients’ excessive tension. Compared with traditional glaucoma surgery, the complication rate of UCP was significantly reduced (12, 20, 22). None of the patients experienced major adverse events, such as severe hypotony or phthisis (the most severe types of complications observed after traditional annular destruction surgery). For the assurance of UCP safety, the innovative HIFU technology has the unique advantage of not only allowing for harmless propagation in living tissue but also allowing energy deposition and tissue heating independent of pigmentation. Therefore, the energy is confined to the distal end of the ciliary body, producing a controlled thermal effect on the target organ, where pigmentation may be highly variable (23, 24). In the eye, ultrasound can heat tissue at any depth or location with precise temperature control. The volume of the treated tissue can be appropriately adjusted, and the integrity of the adjacent tissue can be preserved, therefore minimizing surgical complications (25). However, some patients still experienced complications, such as conjunctival hyperemia, corneal edema, aqueous flare, and hyphema, all of which gradually disappeared within a month and did not require interventional therapy. One patient developed a mild anterior chamber inflammatory reaction with very limited signs of reaction; this also healed within a month. We performed a postoperative UBM review and found no evidence of scleral thinning or damage or adjacent tissue destruction, but some inflammatory mediators may have been synthesized and released after surgery (26, 27). Minor complications seem to be unavoidable, but their incidence was significantly reduced in this study compared to traditional surgery and did not affect the surgical outcomes. Furthermore, UCP carries little potential threat to vision. The patients’ visual acuities did not fluctuate significantly compared with their preoperative measurements, with the exception of one patient, who developed vision loss and astigmatism 1 year after surgery (28). This patient was found to have cataracts before the surgery, and the visual acuity may have been related to the cataract progression. This reduction in complications improves the safety profile of UCP, indicating that UCP is a more gentle and well-tolerated treatment.

In order to better understand the overall efficacy of UCP, we analyzed the patients’ quality of life. We scored various survival indicators and compared the values before and after surgery (29, 30). The index score trends varied among the 7 different indicators. The largest change was in anxiety levels. A lower anxiety score indicated a higher level of anxiety. The patients’ anxiety levels were very high before their surgeries, as reflected by the average score of 32.6 ± 21.1. However, their anxiety was significantly relieved on the first day after surgery and greatly reduced 1 week after surgery. At the last follow-up, the average anxiety score was stable at 61.3 ± 21.7, nearly twice that before the operations. This is a satisfactory result, indicating that UCP had a positive effect on the patients’ psyches.

Large changes were also seen in the psychological wellbeing, self-image, driving, and confidence in health care domains. Although there were no statistically significant changes in these domains on the first postoperative day, the scores were significantly improved 1 week after surgery. Notably, however, in the driving and confidence in health care domains, the scores began to decrease at the last 2 follow-up visits. This may have been because some patients felt good after the treatment and were unwilling to review frequently according to the follow-up standards. There were also some patients who reduced their medication or stopped taking it altogether because their IOP remained stable, causing the scores to show a downward trend after 6 months.

For the daily life domain, the overall score increase was gradual (i.e., the rate of increase was relatively slow), and it did not reflect a change until 1 month after the operation. This may have been because it took some time for the surgical efficacy to have an impact on the patients’ daily lives. However, UCP did not reduce the burden of treatment for patients. We required the type and quantity of IOP-lowering drugs to be the same for patients before and after surgery, which may have made patients feel the pressure of treatment. Overall, UCP improved the patients’ quality of life to a certain extent and helped them to resist the negative emotions brought about by moderate glaucoma.

In this study, we also conducted animal experiments. Through the in-depth exploration of the tissue, we can more clearly understand the working effect of HIFU. In immunofluorescence sections, the ciliary body regions treated with HIFU showed less AQP4 content. The degrees of pigmentation in the pigmented and non-pigmented epithelial cell layers decreased significantly compared with the control group, and some epithelial cells were removed. However, there was no significant difference in the expression levels of AQP4 in the ciliary muscle between the two groups; it was retained in both groups (Figures 4E,H). Only a small amount of AQP4 was lost in the epithelial cell layer in the UCP group. This is because the arc-shaped annular probe used in the operation can accurately locate the ciliary process while focusing the ultrasound on the local area, forming a small thermal coagulation range. Therefore, the maximum damage is always in the ciliary process and dose not injure the ciliary muscle or other adjacent tissues; this reduces the secretion of aqueous humor while retaining the integrity of the blood-aqueous barrier (31, 32). To clarify the extent of the tissue damage, we also analyzed the internal structural changes of the epithelial cells. The penetration of HIFU can produce mild and sustained damage to epithelial cells, resulting in cell degeneration or necrotic shedding (18). Through transmission electron microscopy, we observed that the thermal effect of ultrasonic energy conversion triggered an inflammatory response in the cell in the UCP group; the cells were edematous and the chromatin in the nuclei was condensed and shrunken. At the same time, some organelles, such as the mitochondria and ribosomes, were also damaged. The destruction of epithelial cells weakened the secretory function of the ciliary body, and the production of aqueous humor was reduced, further validating the clinical follow-up results.

Some limitations apply to the present study. Its small sample size and high long-term loss to follow-up rate constitute the limitations of this study, and further prospective randomized clinical trials are needed to confirm the long-term efficacy and safety of the surgery. At present, studies have shown that repeated UCP treatment can be performed, but the optimal time and frequency of repeated treatment remains to be elucidated. More extensive research will be carried out in this direction in the future.

## Conclusion

In conclusion, UCP is an exciting innovation. With the help of computers, surgery can be implemented simply and quickly, which shortens the learning curve for surgeons and minimizes the surgical risk. This approach, which is not completely dependent on surgeons, effectively improves the safety of surgery for moderate glaucoma by weakening a functional, rather than destructive, approach regarding the ciliary body. This study should improve confidence regarding the use of UCP for the treatment of moderate glaucoma, as it indicates that the thermal coagulation effect of HIFU on the ciliary body is an effective method to reduce IOP and control the progression of the disease.

## Data availability statement

The original contributions presented in this study are included in the article/supplementary material, further inquiries can be directed to the corresponding author.

## Ethics statement

This animal study was reviewed and approved by the Medical Ethic Committee, the First Affiliated Hospital of Anhui Medical University.

## Author contributions

R-XW: conceptualization, methodology, formal analysis, writing—original draft, data curation, and visualization. NL: investigation, validation, and data curation. X-YC: validation, writing—review, supervision, project administration, and funding acquisition. All authors contributed to the article and approved the submitted version.
